# Supplementary figures and images for: S-acylation of NLRP3 provides a nigericin sensitive gating mechanism that controls access to the Golgi
Source: eLife. 2024 Sep 12;13:RP94302. doi: 10.7554/eLife.94302 (PMC11392533; doi:10.7554/eLife.94302)

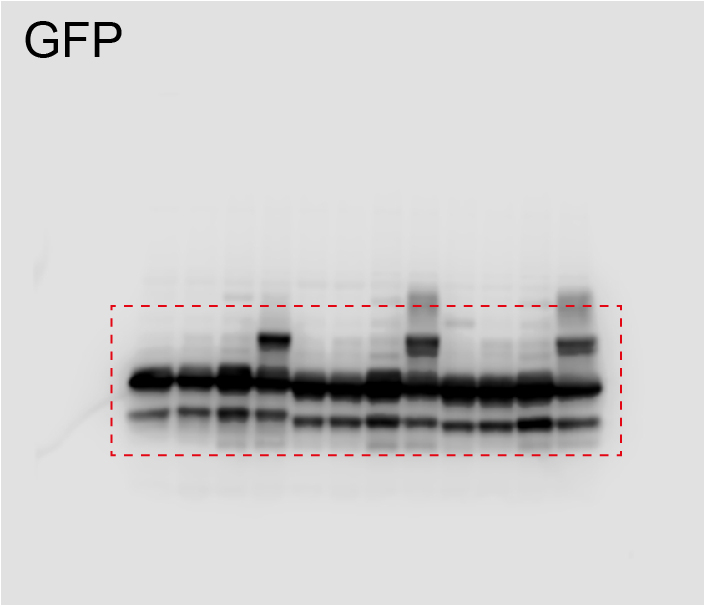

Supplement: Figure 2—figure supplement 1—source data 1. [file elife-94302-fig2-figsupp1-data1.zip › Figure_2_FS1H/Figure_2_FS1H_GFP.jpg]

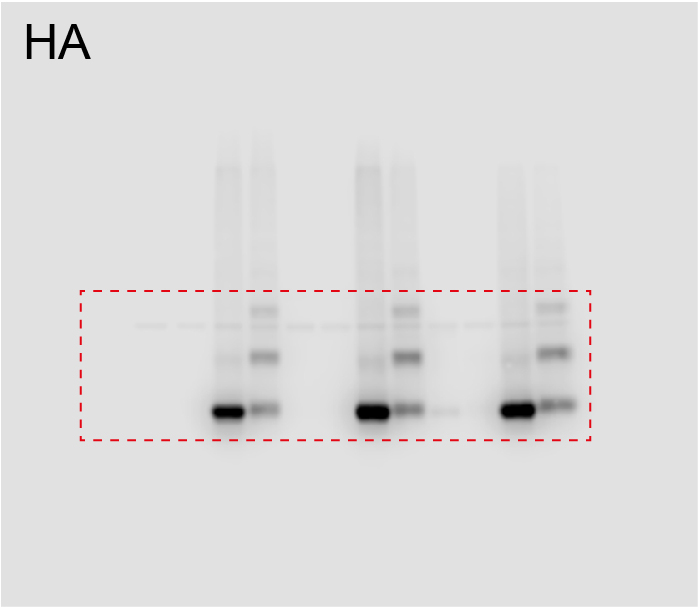

Supplement: Figure 2—figure supplement 1—source data 1. [file elife-94302-fig2-figsupp1-data1.zip › Figure_2_FS1H/Figure_2_FS1H_HA.jpg]

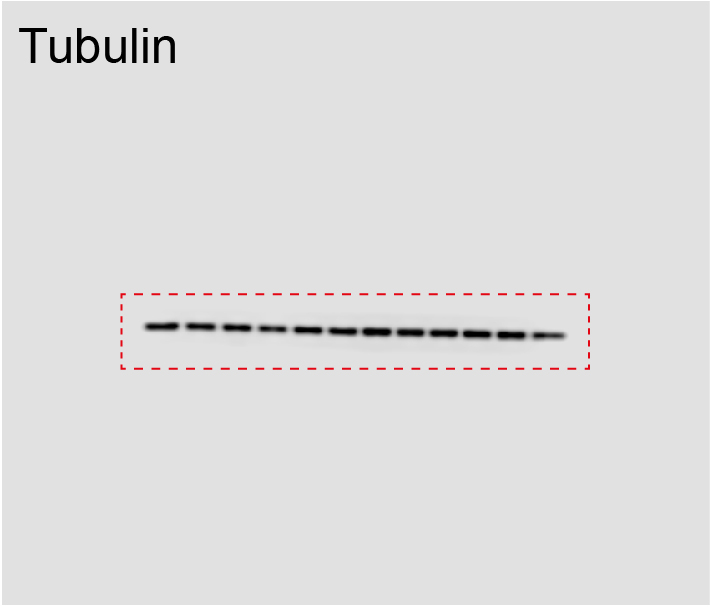

Supplement: Figure 2—figure supplement 1—source data 1. [file elife-94302-fig2-figsupp1-data1.zip › Figure_2_FS1H/Figure_2_FS1H_Tubulin.jpg]

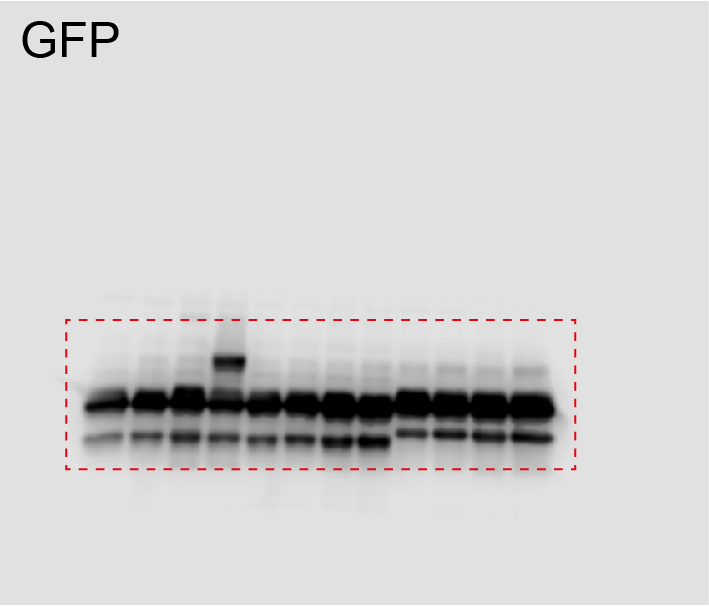

Supplement: Figure 4—source data 1. [file elife-94302-fig4-data1.zip › Figure_4G_GFP.jpg]

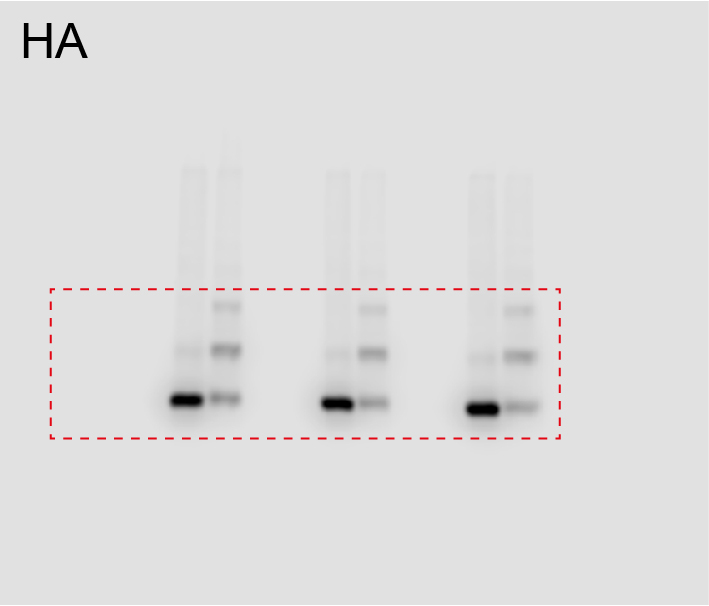

Supplement: Figure 4—source data 1. [file elife-94302-fig4-data1.zip › Figure_4G_HA.jpg]

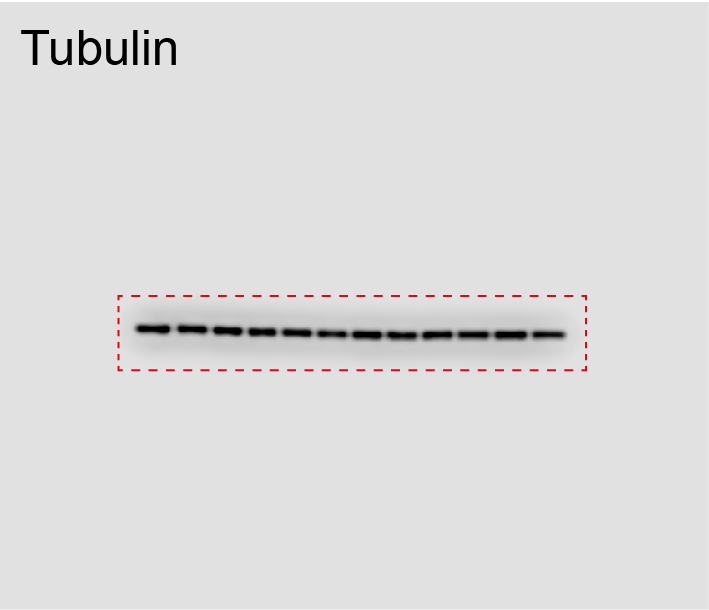

Supplement: Figure 4—source data 1. [file elife-94302-fig4-data1.zip › Figure_4G_Tubulin.jpg]

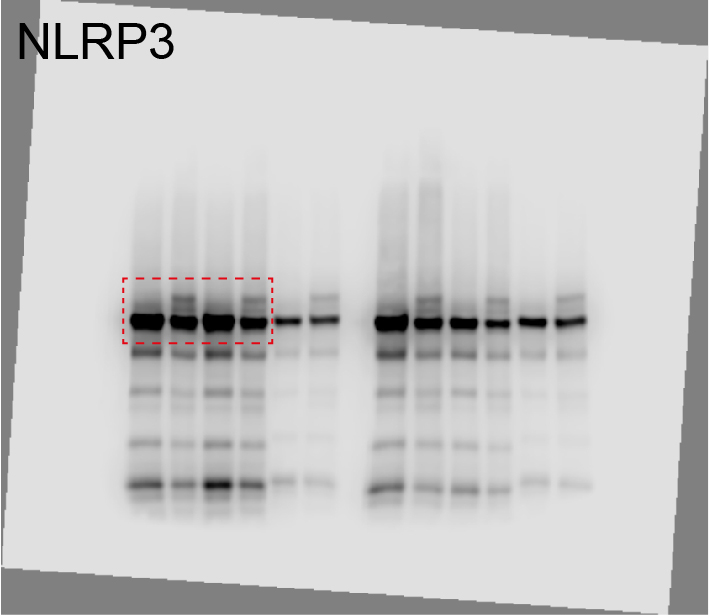

Supplement: Figure 4—source data 1. [file elife-94302-fig4-data1.zip › Figure_4C_NLRP3.jpg]

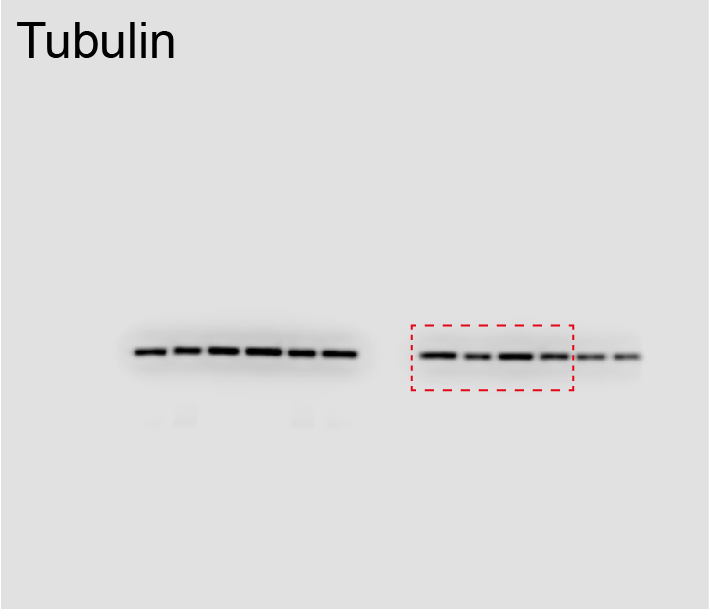

Supplement: Figure 4—source data 1. [file elife-94302-fig4-data1.zip › Figure_4C_Tubulin.jpg]

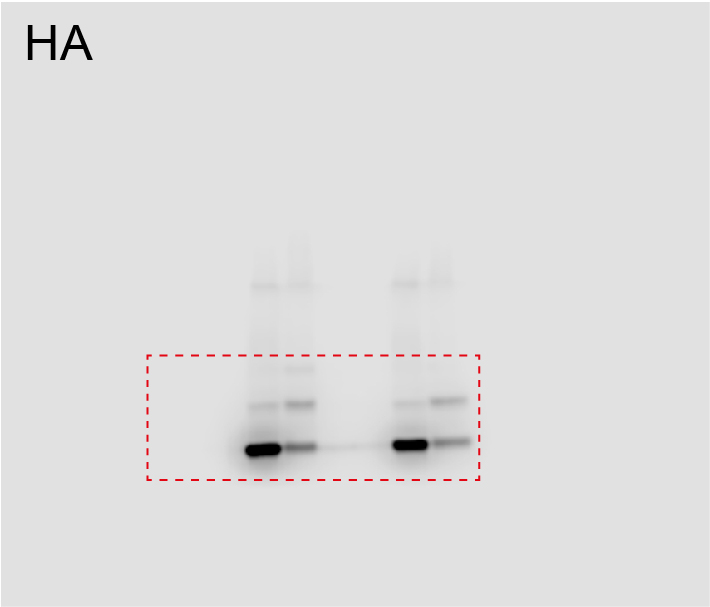

Supplement: Figure 4—source data 1. [file elife-94302-fig4-data1.zip › Figure_4B_HA.jpg]

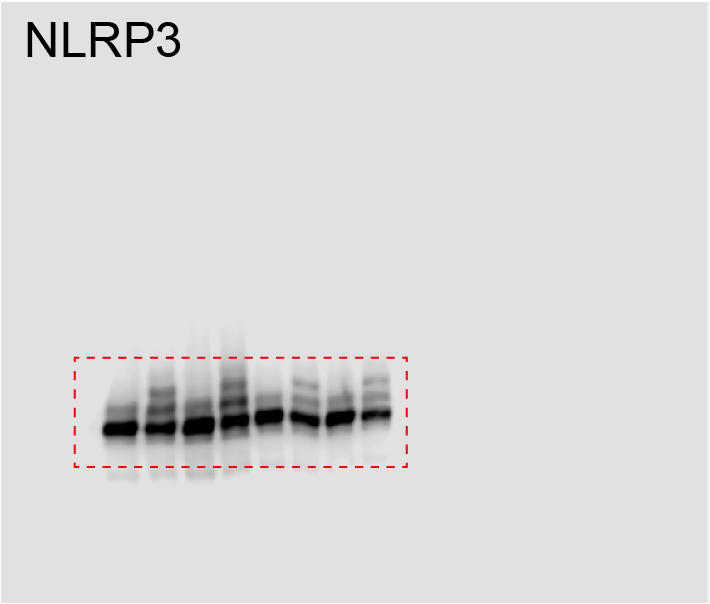

Supplement: Figure 4—source data 1. [file elife-94302-fig4-data1.zip › Figure_4B_NLRP3.jpg]

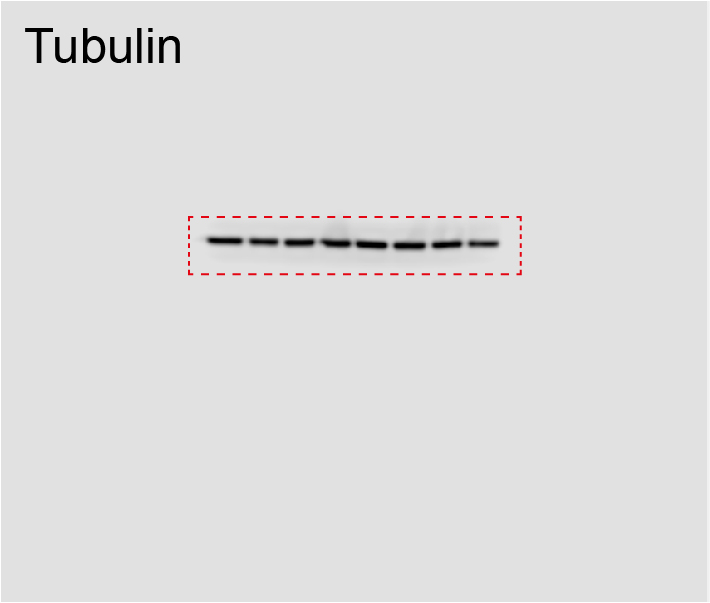

Supplement: Figure 4—source data 1. [file elife-94302-fig4-data1.zip › Figure_4B_Tubulin.jpg]

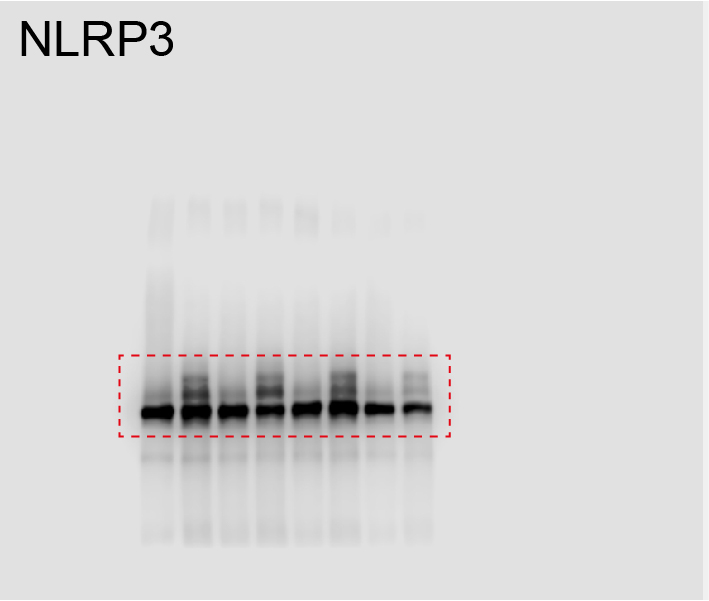

Supplement: Figure 4—source data 1. [file elife-94302-fig4-data1.zip › Figure_4A_NLRP3.jpg]

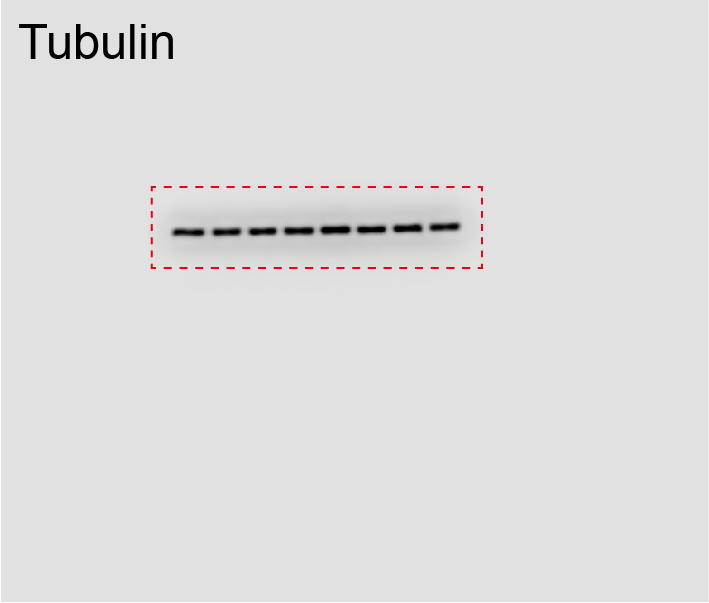

Supplement: Figure 4—source data 1. [file elife-94302-fig4-data1.zip › Figure_4A_Tubulin.jpg]
